# The comparison between weekly and three-weekly cisplatin delivered concurrently with radiotherapy for patients with postoperative high-risk squamous cell carcinoma of the oral cavity

**DOI:** 10.1186/1748-717X-7-215

**Published:** 2012-12-18

**Authors:** Din-Li Tsan, Chien-Yu Lin, Chung-Jan Kang, Shiang-Fu Huang, Kang-Hsing Fan, Chun-Ta Liao, I-How Chen, Li-Yu Lee, Hung-Ming Wang, Joseph Tung-Chieh Chang

**Affiliations:** 1 Department of Radiation Oncology, Chang Gung Memorial Hospital, 5, Fu-Shin St. Kwei-Shan, Tao-Yuan Hsien, Taiwan; 2 Department of Otorhinolaryngology, Chang Gung Memorial Hospital, Taoyuan, 333, Taiwan; 3 Department of Pathology, Chang Gung Memorial Hospital, Taoyuan, 333, Taiwan; 4 Divsion of Hematology/Oncology, Department of Internal Medicine, Chang Gung Memorial Hospital, Taoyuan, 333, Taiwan; 5 Graduate Institute of Clinical Medical Science, Chang Gung University, Taoyuan, 333, Taiwan; 6 College of Medicine, Chang Gung University, Taoyuan, 333, Taiwan

**Keywords:** Oral cavity cancer, Concurrent chemoradiotherapy, Acute toxicity, Compliance, Health-related quality of life

## Abstract

**Background:**

The aim of this study was to compare the outcomes of postoperative adjuvant concomitant chemoradiotherapy using two different schedules of cisplatin for patients with high-risk oral squamous cell carcinoma (OSCC).

**Methods:**

From Feb. 2008 to Aug. 2010, 55 patients with high-risk OSCC were included in this study. Patients were randomized into treatment groups that either received 100 mg/m^2^ cisplatin once every 3 weeks (arm A) or 40 mg/m^2^ cisplatin once per week (arm B). All patients were irradiated with 66 Gy in 33 fractions.

**Results:**

Of the 50 eligible patients, 26 were assigned to arm A, and 24 were assigned to arm B. Both groups of patients received the same mean doses of radiotherapy and cisplatin. However, 88.5% of patients in arm A and 62.5% of those in arm B (p = 0.047) received ≥ 200 mg/m^2^ of cisplatin in total. The overall toxicity was significantly greater in arm B (p = 0.020), and all of the grade 4 toxicities occurred in patients in arm B.

**Conclusions:**

Three-weekly high-dose cisplatin treatment showed higher compliance, and lower acute toxicity compared to weekly low-dose cisplatin treatment.

## Background

Surgery is the primary therapeutic treatment options for locally advanced oral cavity squamous cell carcinoma (OSCC). Patients with pathologically documented extracapsular spreading (ECS) of the involved lymph node (LN), a positive surgical margin, or those with LN staging ≥ N2 are at high risk for therapeutic failure. Concurrent chemoradiotherapy (CCRT) improves overall survival (OS), disease-free survival (DFS), and locoregional control (LRC) and has become the standard treatment for postoperative high-risk squamous cell carcinomas of the head and neck (SCCHN) since the Radiation Therapy Oncology Group (RTOG) and European Organisation for Research and Treatment of Cancer (EORTC) randomized phase III trials [1-3].

A 100 mg/m^2^ dose of cisplatin administered once every 3 weeks concurrently with radiotherapy (RT) is a commonly recommended treatment regimen during CCRT for SCCHN. Its high emetic potential, neurotoxicity, and ototoxicity demand further efforts be made towards improving its therapeutic and toxicity profiles. Several other chemotherapy regimens for CCRT use a different schedule of cisplatin to improve compliance and the toxicity profile. Among these regimens, weekly cisplatin doses ranging from 30 to 40 mg/m^2^ are used most widely in the radical and adjuvant settings [4-6].

A 40 mg/m^2^ dose of cisplatin administered once per week has been widely used in CCRT for cervical cancer, as it is effective and has a relatively low toxicity [7-10]. A randomized phase III trial that evaluated the weekly administration of 40 mg/m^2^ cisplatin plus RT versus RT alone for the treatment of nasopharyngeal cancer also demonstrated favorable outcomes in patients with an advanced T-stage treated by CCRT [11,12]. Several studies have compared the weekly and 3-weekly cisplatin CCRT regimens in patients with SCCHN, and the results have been inconclusive [13-15]. However, no randomized controlled trials have compared the efficacy and toxicity profiles of these two schedules of cisplatin as part of CCRT for SCCHN.

**Figure 1:**
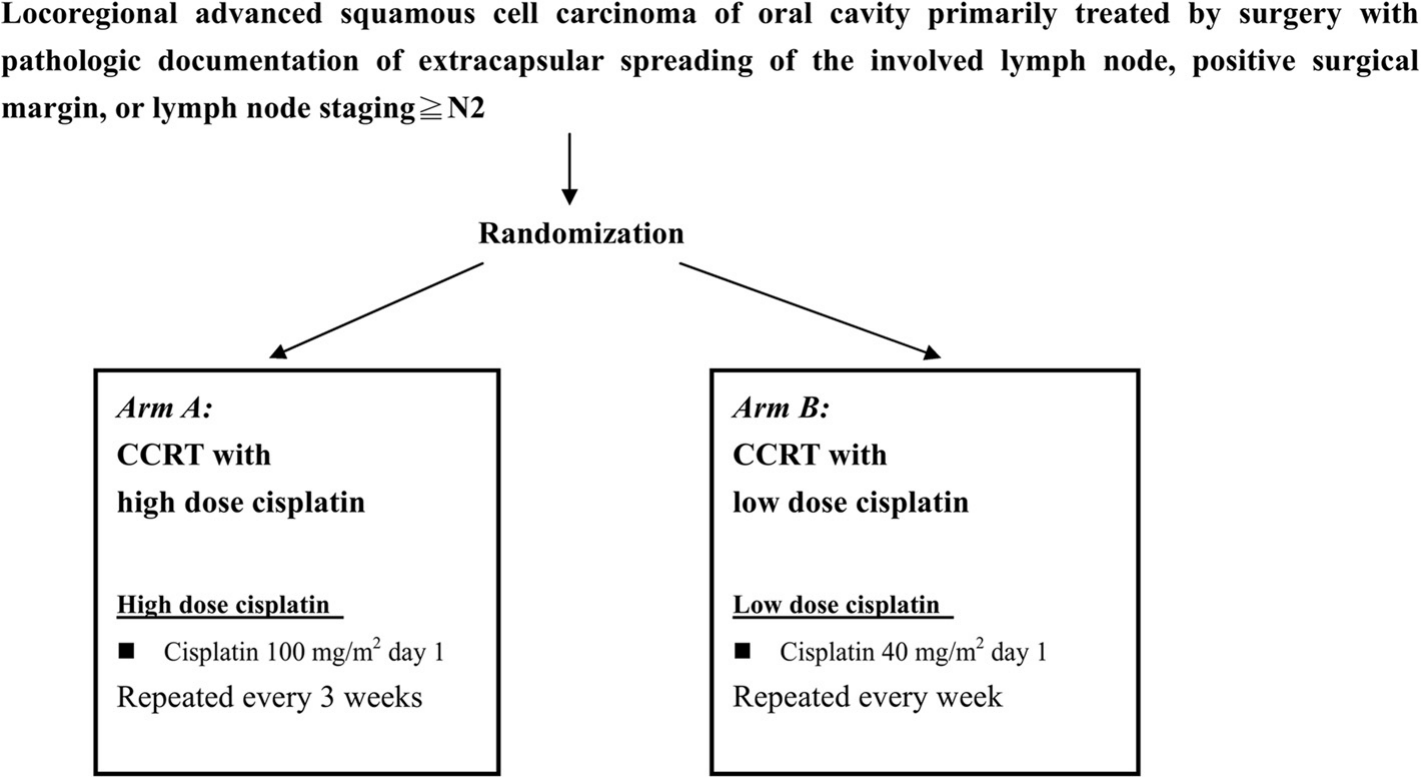
**Study design.** Patients with postoperative high-risk squamous cell carcinoma of the oral cavity were randomized to receive either cisplatin at 100 mg/m^2^ once every 3 weeks (arm **A**) or cisplatin at 40 mg/m^2^ once per week (arm **B**). *Abbreviations:* CCRT = concurrent chemoradiotherapy.

We therefore designed a phase III randomized trial to investigate the efficacy and toxicity profile of postoperative adjuvant CCRT using weekly versus three-weekly cisplatin for patients with advanced OSCC and pathologic risk factors for therapeutic failure (Figure 1). The study endpoints included OS, LRC, and the distant control rate for the postoperative adjuvant CCRT patients treated with either of the two cisplatin regimens. This preliminary report aimed to compare the compliance, acute toxicities, and health-related quality of life (HRQoL) between the two cisplatin treatment groups.

## Methods

### Study design

This was a phase III randomized study for postoperative adjuvant CCRT that compared three-weekly high-dose and weekly low-dose cisplatin for the treatment of patients with advanced OSCC and pathologic risk factors of therapeutic failure. The study was approved by the Institutional Review Board for Human Research. The trial was a superiority design in favor of better efficacy of weekly low dose cisplatin regimen. It was designed to detect an absolute increase in progression-free survival of 15% (from 40% to 55% at 3 years) with a two-sided 5% significance level and a statistical power of 80%. The study required the randomization of 338 eligible patients, and 371 patients were scheduled to account for an expected rate of ineligibility and a loss to follow-up of up to 10%. However, due to slow recruitment, the trial was ended after only 55 patients were recruited over 30 months.

### Patient selection

Prior to the onset of therapy, the patients were thoroughly informed about all aspects of the study and regulatory requirements that needed be satisfied for informed consent. All screening tests, examinations and procedures were completed prior to the initial dose of CCRT. The eligible patients were between 18 and 70 years old and had an Eastern Cooperative Oncology Group (ECOG) performance status (PS) of 0–2 and adequate bone marrow, liver, and renal function. All patients needed to have histologically confirmed primary OSCC, pathologic documentation for ECS of the involved LN, a positive surgical margin, or LN staging ≥ N2. The patients were staged according to the staging criteria of the 2002 American Joint Committee on Cancer (AJCC, 6th edition).

Patients were excluded if they had suspected distant metastatic lesions, as detected by imaging techniques, such as magnetic resonance imaging (MRI)/computed tomography (CT) or 2-deoxy-2[F-18]fluoro-D-glucose positron emission tomography (FDG-PET). In addition, patients could not have received prior chemotherapy or RT, and patients with a serious concomitant illness, such as active cardiac disease, severe uncontrolled hypertension, uncontrolled infection, or a history of other head and neck malignancies, were excluded from this study.

### Treatment

All study participants underwent an extensive pretreatment evaluation, which included a medical history, a complete physical examination, a complete blood count and routine blood biochemistry panel, CT or MRI scans of the head and neck, chest radiography, bone scans, liver ultrasonography, and FDG-PET scans. The surgical procedure that all patients received involved the composite resection of the tumor with immediate flap reconstruction and neck dissections.

The patients who were randomized to the three-weekly high-dose cisplatin arm were treated with cisplatin at 100 mg/m^2^ once every 3 weeks (arm A), and the patients who were randomized to the low-dose cisplatin arm were treated with cisplatin at 40 mg/m^2^ once per week (arm B). Radiotherapy was administered using 6 MV photon beams at a conventional fractionation dose of 2 Gy/fraction (fx)/day and 5 days per week. All patients were treated on an out-patient basis and were irradiated with a total dose of 66 Gy.

### Assessment and outcomes

Follow-up visits were continued until Feb. 2011. OS time was calculated as the period between the date of randomization and the date of death. Locoregional recurrence-free survival (LRRFS) time was calculated as the period between the date of randomization and the date of local failure, regional failure, or death. Toxicity was assessed according to the National Cancer Institute (NCI) Common Terminology Criteria for Adverse Events (CTCAE) version 3.0. Acute toxicity was defined as toxicity that was noted during the RT or during the following 3 months after the completion of the RT.

The patients were invited to complete the HRQoL questionnaire as well as demographic and clinical questionnaires during the course of treatment and follow-up. We used the Chinese version of the Functional Assessment of Cancer Therapy - Head and Neck (FACT-H&N) questionnaire for the HRQoL survey.

### Statistical analysis

Commercial statistical software (SPSS 11.0; SPSS, Chicago, IL) was used for the statistical analysis. The descriptive statistics were summarized using frequencies, percentages, means, medians, standard deviations, and ranges. The variables that could have affected the outcomes were evaluated using a chi-squared test, an independent *t*-test, or Fisher’s exact test, as appropriate. The Kaplan-Meier method was used to analyze survival times, and the log-rank test was used to test equality between groups. For all analyses, p < 0.05 (two-tailed) was considered statistically significant.

## Results

Between Feb. 2008 and Aug. 2010, 55 patients were enrolled in this study. However, after reviewing the patient data, only 50 patients were validated for inclusion in the analysis. Of the 5 patients excluded, 4 did not completely satisfy the inclusion criteria, as 2 patients had previously been treated for primary head and neck malignancies, 1 patient had a pathology indicative of myoepithelial carcinoma instead of SCCHN, and 1 patient was found to have no major pathologic risk factors, such as ECS of the involved LN, a positive surgical margin, or LN staging ≥ N2. The remaining patient was taken into custody after randomization, which meant he was unable to start treatment and was excluded from the analysis. Of the 50 eligible patients, 26 patients were assigned to arm A (cisplatin 100 mg/m^2^ every 3 weeks), and 24 patients were assigned to arm B (cisplatin 40 mg/m^2^ every week). The patients in these treatment arms did not differ in terms of gender, age, pT, pN, or stage (see Table 1).

**Table 1:** The demographic and oncological characteristics

**Patient characteristics**	**Arm A (cisplatin 100 mg/m^2^)**	**Arm B (cisplatin 40 mg/m^2^)**	**p**
	**N = 26**	**N = 24**	
Gender			
Male	25 (96.2%)	23 (95.8%)	1.000
Female	1 (3.8%)	1 (4.2%)	
Age (years old)			
Mean (range)	49.2 (33–63)	49.0 (32–65)	0.941
pT			
pT1/2	14 (53.8%)	11 (45.8%)	0.778
pT3/4	12 (46.2%)	13 (54.2%)	
pN			
pN0	0 (0%)	2 (8.3%)	
pN1	5 (19.2%)	8 (33.3%)	
pN2	21 (80.8%)	14 (58.3%)	
Stage			
Stage II	0 (0%)	1 (4.2%)	
Stage III	3 (11.5%)	4 (16.7%)	
Stage IV	23 (88.5%)	19 (79.2%)	
Differentiation			
Well	7 (26.9%)	5 (20.8%)	
Moderate	15 (57.7%)	17 (70.8%)	
Poor	4 (15.4%)	2 (8.3%)	
Primary Site			
Buccal	10 (38.5%)	9 (37.5%)	
Tongue	10 (38.5%)	11 (45.8%)	
Gum	5 (19.2%)	3 (12.5%)	
Others	1 (3.8%)	1 (4.2%)	
ECS			
No	5 (19.2%)	9 (37.5%)	0.211
Yes	21 (80.8%)	15 (62.5%)	
Margin			
Negative	25 (96.2%)	20 (83.3%)	0.182
Positive	1 (3.8%)	4 (16.7%)	
Tumor size (mm)			
Mean (range)	31.19 (12–68)	34.13 (12–60)	0.414

*Abbreviations: ECS* = extracapsular spreading.

The treatment characteristics are shown in Table 2. Both groups of patients received the same mean doses of RT and cisplatin, and they also did not differ regarding the inadequate RT (RT dose < 60 Gy) or interrupted RT (RT duration > 8 weeks) rate. However, in terms of adequate cisplatin dose, which was defined as cisplatin ≥ 200 mg/m^2^, more patients in arm A received an adequate cisplatin dose than did patients in arm B. In arm A, 88.5% of patients received an adequate dose of cisplatin, whereas only 62.5% of those in arm B received an adequate dose (p = 0.047).

**Table 2:** Treatment characteristics

**Treatment characteristics**	**Arm A (cisplatin 100 mg/m^2^)**	**Arm B (cisplatin 40 mg/m^2^)**	**p**
	**N = 26**	**N = 24**	
Cisplatin dose (mg/m^2^)			
Mean (range)	208.5 (100–300)	200.4 (80–280)	0.568
Cisplatin ≥ 200 mg/m^2^			
No	3 (11.5%)	9 (37.5%)	0.047*
Yes	23 (88.5%)	15 (62.5%)	
RT dose (cGy)			
Mean (range)	6477.7 (4820–6600)	6250.0 (1400–7200)	0.361
RT dose ≥ 6000 cGy			
No	2 (7.7%)	2 (8.3%)	1.000
Yes	24 (92.3%)	22 (91.7%)	
RT duration (weeks)			
Mean (range)	6.8 (4.6–9.1)	6.6 (1.7–9.0)	0.506
RT duration > 8 weeks			
No	24 (92.3%)	20 (83.3%)	0.409
Yes	2 (7.7%)	4 (16.7%)	

*Abbreviations: RT* = radiotherapy.

* Statistically significant, p < 0.05.

The CCRT toxicities are shown in Table 3. The overall toxicity was significantly greater for patients in arm B (p = 0.020), as all of the grade 4 toxicities were observed in arm B. The cases of grade 4 toxicity included 3 cases of pharyngitis, 2 cases of stomatitis, 1 case of nausea/vomiting, and 1 case of laryngeal edema. The patients in arm B also showed a greater incidence of mucositis, as cases of mucositis ≥ grade 3 were 38.5% of the patients in arm A and 75% in arm B (p = 0.012). The two groups of patients did not differ in terms of hematologic toxicity. We did not observe ≥ grade 3 renal toxicity or ototoxicity, although there were 2 patients with grade 2 renal toxicity (one in each arm).

**Table 3:** Acute toxicity profile

**CCRT toxicity**	**Arm A (cisplatin 100 mg/m^2^)**	**Arm B (cisplatin 40 mg/m^2^)**	**p**
	**N = 26**	**N = 24**	
Overall toxicity			
Grade 2	5 (19.2%)	2 (8.3%)	0.020*
Grade 3	21 (80.8%)	16 (66.7%)	
Grade 4	0 (0%)	6 (25.0%)	
Non-hematologic			
Mucositis			
< Grade 3	16 (61.5%)	6 (25.0%)	0.012*
≥ Grade 3	10 (38.5%)	18 (75.0%)	
Pharyngitis			
< Grade 3	12 (46.2%)	11 (45.8%)	1.000
≥ Grade 3	14 (53.8%)	13 (54.2%)	
Stomatitis			
< Grade 3	12 (46.2%)	11 (45.8%)	1.000
≥ Grade 3	14 (53.8%)	13 (54.2%)	
Laryngeal edema			
< Grade 3	23 (88.5%)	23 (95.8%)	0.611
≥ Grade 3	3 (11.5%)	1 (4.2%)	
Dermatitis			
< Grade 3	24 (92.3%)	22 (91.7%)	1.000
≥ Grade 3	2 (7.7%)	2 (8.3%)	
Nausea/vomiting			
< Grade 3	23 (88.5%)	19 (79.2%)	0.456
≥ Grade 3	3 (11.5%)	5 (20.8%)	
Hematologic			
Anemia			
< Grade 3	25 (96.2%)	23 (95.8%)	1.000
≥ Grade 3	1 (3.8%)	1 (4.2%)	
Leukopenia			
< Grade 3	26 (100%)	21 (87.5%)	0.103
≥ Grade 3	0 (0%)	3 (12.5%)	
Neutropenia			
< Grade 3	26 (100%)	23 (95.8%)	0.480
≥ Grade 3	0 (0%)	1 (4.2%)	
Thrombocytopenia			
< Grade 3	26 (100%)	24 (100%)	NS
≥ Grade 3	0 (0%)	0 (0%)	

*Abbreviations: CCRT* = concurrent chemoradiotherapy; *NS* = no statistics were computed.

* Statistically significant, p < 0.05.

**Figure 2:**
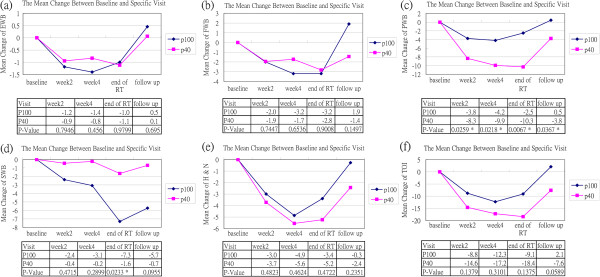
**Health-related quality of life changes.** Values represent the mean changes between the scores at baseline and at each specific visit. **(a)** physical well-being (PWB), **(b)** social well-being (SWB), **(c)** physical well-being (PWB), **(d)** social well-being (SWB), **(e)** head and neck (H&N) subscale, **(f)** trial outcome index (TOI). *Statistically significant, p < 0.05.

The HRQoL data were collected using the FACT-H&N at each clinic visit during the course of treatment and follow-up. We chose the following 5 time points for analysis based on the typical fluctuations in quality of life that are experienced during radiotherapy and chemotherapy: baseline (no later than 1 week after the start of treatment), 2 weeks after the start of treatment (± 1 week), 4 weeks after the start of treatment (± 1 week), the end of RT (± 1 week), and follow-up (3 months after the end of RT, ± 1 month). We subtracted the results at baseline from those at each time point for the eligible patients and compared the differences in HRQoL decrease between groups. The result is shown in Figure 2. The emotional well-being (EWB), functional well-being (FWB), and FACT head and neck (H&N) subscale scores were not different between the two arms (data not shown, see additional files). For physical well-being (PWB), the scores of patients in arm B decreased more significantly following treatment than did those of the patients in arm A. Moreover, these differences were significant for all time points analyzed. For social well-being (SWB), the scores of patients in arm A decreased more significantly than did those of the patients in arm B at the end of the RT. The Trial Outcome Index (TOI) is a combined scale that consists of PWB, FWB, and H&N subscales. The TOI scores of patients in arm B decreased slightly more than those for patients in arm A, but this difference did not reach significance. However, there was a trend towards a better recovery of the TOI score for patients in arm A at follow-up.

**Figure 3:**
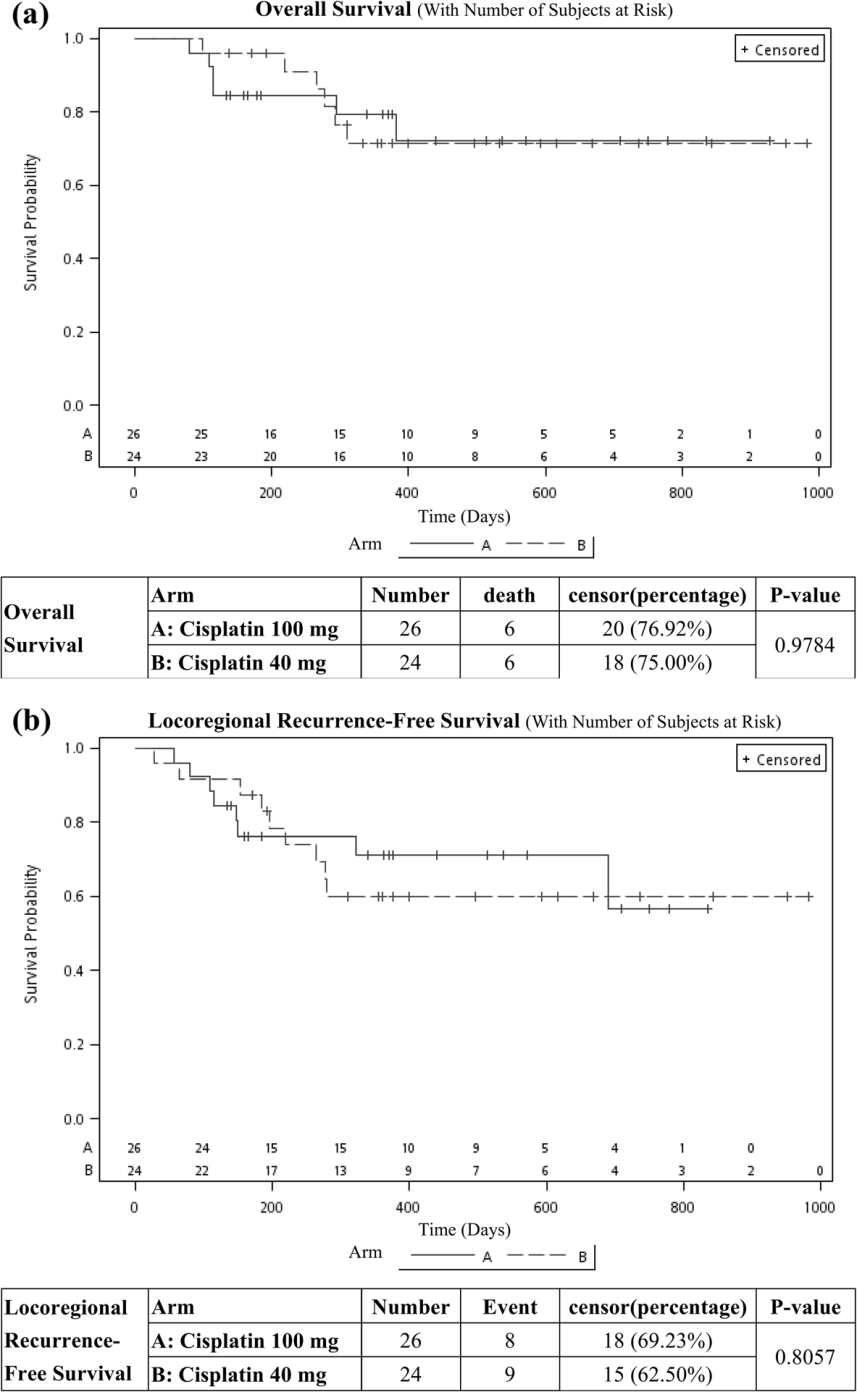
**Preliminary results.** After a median follow-up period of 12.0 months, the overall survival (OS) and locoregional recurrence-free survival (LRRFS) were not different between the two groups. **(a)** The 1-year OS was 79.3% and 71.6% for patients in arm **A** and arm **B**, respectively (p = 0.978). **(b)** The 1-year LRRFS was 71.1% and 60.0% for patients in arm **A** and arm **B**, respectively (p = 0.806).

After a median follow-up period of 12.0 months (range 2.7 to 32.8 months), the preliminary OS and LRRFS were not different between the two groups (Figure 3). The 1-year OS was 79.3% and 71.6% for patients in arm A and arm B, respectively (p = 0.978), and the 1-year LRRFS was 71.1% and 60.0% for patients in arm A and arm B, respectively (p = 0.806).

## Discussion

In our experience, the 5-year rates for patients with tongue and buccal carcinomas have been as follows: local and neck control, approximately 85%; distant metastasis, 8%–14%; and disease-free survival, 70%–72% [16]. From our previous analysis of 201 patients with advanced squamous cell carcinoma of the oral tongue, the 3-year OS and recurrence-free survival (RFS) rates were 48% and 50.8%, respectively. If ECS was present, CCRT was shown to significantly improve survival (3-year RFS with ECS and with CCRT = 48.2% vs. without CCRT = 15%) [17].

Concurrent administration of 100 mg/m^2^ cisplatin once every 3 weeks and RT is recommended as the standard regimen for adjuvant CCRT for SCCHN because two large-scale randomized trials used this regimen [1,2]. However, alternatives, such as weekly administration during RT, may show similar efficacy, and less toxicity. Schedules that deliver cisplatin in smaller doses on a more frequent basis may be preferable to cyclical bolus administration for two reasons. First, more frequent administration could provide radiosensitizing chemotherapy as a larger proportion of the administered RT dose. Second, smaller individual doses of cisplatin may lead to less chemotherapy-induced morbidity without compromising efficacy [18]. Marcu et al. have done extensive modeling studies and review on cisplatin radio-sensitization [19,20]. Their result also support our hypothesis that with more frequent administration of low dose cisplatin, the radio-sensitization effect can be best improved. They designed a model to simulate the combined cisplatin-radiotherapy treatment with the emphasis on time sequencing and scheduling of drug and radiation. The model showed that daily administration of cisplatin led to a 35% improvement of tumor control as compared to radiation alone, while weekly cisplatin has improved radiotherapy by only 6% [19]. Their review also found daily low-dose cisplatin performed in 6 out of the 16 trials demonstrated increased tumor control with less toxicity as compared to weekly high-dose drug delivery [20]. Maybe further optimize the treatment schedule of combined chemoradiotherapy, such as daily administration of cisplatin, will be of interest in our future studies. Compliance is another significant problem with the standard cisplatin CCRT regimen. A minimum cumulative dose of 200 mg/m^2^ during the course of irradiation is generally accepted [21]. In the RTOG 9501 study, 61% of patients received all 3 planned cycles of cisplatin, 23% received 2 cycles, 13% received 1 cycle, and 2% received no chemotherapy [2]. In the EORTC 22931 study, compliance to chemotherapy also decreased according to the number of courses delivered, as the first, second, and third cycles were administered to 88%, 66%, and 49% of patients, respectively [1]. The weekly 40 mg/m^2^ dose of cisplatin is thought to be more easily administered than cisplatin at a dosage of 100 mg/m^2^ every 3 weeks.

To the best of our knowledge, this is the first randomized trial comparing 2 different cisplatin CCRT approaches for patients with SCCHN. In the current study, no differences were found between groups in terms of RT compliance. Although no statistically significant difference in the mean cisplatin dose was noted, significantly more patients in arm A received a cumulative dose of 200 mg/m^2^ than did those in arm B. An inadequate dose of cisplatin could be associated with a worse long-term treatment result, although various studies support the use of weekly low-dose cisplatin. In a retrospective study by Ho et al., the dose intensity of cisplatin was compared for one weekly and two different 3-weekly regimens [14]. Both the mean cisplatin dose and the cumulative dose achieved were not significantly different between the weekly and the lower-dose 3-weekly group. However, no patients in the higher-dose 3-weekly group received the full 3 cycles of cisplatin.

Ho et al. reported similar toxicities between the weekly and 3-weekly groups [14]. The patients treated with 3-weekly cisplatin seemed to suffer more grade 3 radiation dermatitis (56% vs. 26%), but this difference was not significant. Uygun et al. reported that grade 3–4 toxic events were observed in 53.3% of the patients treated with 3-weekly cisplatin and 40% of those treated with weekly cisplatin, but this difference was also not significant [15]. However, Geeta et al. suggested that 3-weekly cisplatin is less toxic than weekly treatment, as their weekly cisplatin schedule resulted in a higher rate of severe mucositis, which was significant in both the univariate and multivariate analyses [13]. In our study, the patients in arm B (low-dose weekly cisplatin) suffered more severe mucositis than did the patients in arm A. The overall toxicity was also greater in arm B, as all grade 4 toxicities were observed for patients in arm B. One possible reason for this enhanced toxicity in arm B may be the lower adherence of these patients to the treatment protocol. Three patients in arm B received 5-fluorouracil in addition to cisplatin, and another patient received RT at a total dose of 72 Gy. However, even with the exclusion of these 4 patients, the mucositis and overall toxicity were still significantly worse for patients in arm B than arm A. Forced hydration with normal saline 500 ml infusion over 2 hours before and after cisplatin infusion is only mandatory in arm A. It is possible that the hydration and post-chemotherapy care of patients may also account for some difference in toxicity.

A comparison of the HRQoL between the weekly low-dose and 3-weekly high-dose cisplatin CCRT has not been previously reported. Thus, our study appears to be the first to examine the differences in HRQoL between these two groups of patients. Our results indicated that the PWB of patients in arm B decreased more significantly (i.e., became worse) than that of patients in arm A at each time point analyzed. PWB represents the HRQoL subscale that is most closely related to treatment toxicity, which means that the greater decrease in PWB observed also reflects the greater level of acute toxicity in arm B. However, the patients in arm A showed a lower level of SWB at the end of the RT, which is hard to explain clinically. It is possible that patients in arm A did not exhibit many signs of toxicity, which caused them to receive less care and support than they needed.

The present study had certain notable limitations. First, although we planned to enroll a total of 371 patients, we were only able to enroll 55 patients over the course of 3 years. With only 50 validated patients for the analysis, the statistical power may not have been sufficient. Second, this study was considered an early report regarding only compliance, acute toxicity, HRQoL, and preliminary survival results. However, we do plan to follow these patients and report additional details regarding their treatment results, such as OS, LRC, and DFS when the data become available. Gupta et al. were also initiating a randomized trial of weekly versus 3-weekly cisplatin in locoregionally advanced SCCHN, we will be anticipating the results [4].

## Conclusions

Three-weekly high-dose cisplatin showed high compliance, low acute toxicity, and better PWB compared to weekly low-dose cisplatin and is feasible for administration in hospital out-patient settings.

## Abbreviations

(OSCC): Oral cavity squamous cell carcinoma; (ECS): Extracapsular spreading; (LN): Lymph node; (CCRT): Concurrent chemoradiation; (OS): Overall survival; (DFS): Disease-free survival (DFS); (LRC): Locoregional control; (SCCHN): Squamous cell carcinomas of the head and neck; (RTOG): Radiation Therapy Oncology Group; (EORTC): European organisation for research and treatment of cancer; (HRQoL): Health-related quality of life; (ECOG): Eastern cooperative oncology group; (PS): Performance status; (AJCC): American joint committee on cancer; (MRI): Magnetic resonance imaging; (CT): Computed tomography; (PET): 2-deoxy-2[F-18]fluoro-D-glucose positron emission tomography; (RT): Radiotherapy; (fx): Fraction; (LRRFS): Locoregional recurrence-free survival; (NCI): National cancer institute; (CTCAE): Common terminology criteria for adverse events; (FACT): Functional assessment of cancer therapy; (EWB): Emotional well-being (EWB); (FWB): Functional well-being (FWB); (H&N): Head and neck (H&N); (PWB): Physical well-being; (SWB): Social well-being; (TOI): Trial outcome index; (RFS): Recurrence-free survival.

## Competing interests

The authors declare that they have no competing interests.

## Authors’ contributions

DLT, HMW and JTCC prepared the study concept and design. DLT did the major manuscript writing. HMW and JTCC did the major revision of the manuscript. LYL made the major contribution for tissue processing. CYL, CJK, SFH,KHF, CTL and IHC participated in the clinical data interpretation. CYL, CJK, SFH, KHF, CTL, IHC, HMW and JTCC treated the patients and did the data collection. All authors read and approved the final manuscript.
